# Improving shared decision-making in a clinical obstetric ward by using the three questions intervention, a pilot study

**DOI:** 10.1186/s12884-018-1921-z

**Published:** 2018-07-04

**Authors:** S. W. E. Baijens, A. G. Huppelschoten, J. Van Dillen, J. W. M. Aarts

**Affiliations:** 1 Gynaecology and Obstetrics Department, Meander Medical Hospital, Maatweg 3, 3813 TZ Amersfoort, the Netherlands; 2 Gynaecology and Obstetrics Department, Jeroen Bosch Hospital, Henri Dunantstraat 1, 5223 GZ ’s-Hertogenbosch, the Netherlands; 3 Gynaecology and Obstetrics Department, Radboudumc, Geert Grooteplein Zuid, 10 6525 GA Nijmegen, the Netherlands

**Keywords:** Shared decision making, Inpatient department, Three questions intervention, Obstetrics

## Abstract

**Background:**

Shared decision-making (SDM) is an important aspect of modern health care. Many studies evaluated different interventions to improve SDM, however, none in an inpatient clinical setting. A tool that has been proven effective in an outpatient department is the three questions intervention. These questions are created for patients to get optimal information from their medical team and to make an informed medical decision. In this study, we evaluated the feasibility and effectiveness of this simple intervention on SDM in the obstetric inpatient department of a university hospital in the Netherlands.

**Method:**

This is a clinical pilot before and after study, using mixed methods with quantitative and qualitative data collection. The three questions were stated on a card; (i.e. 1) What are my options; 2) What are the possible benefits and harms of those options; 3) How likely are each of those benefits and harms to happen to me?). The study period lasted 6 weeks in which all patients admitted to the obstetric ward were asked to participate in the study. In the first 3 weeks patients did not receive the three questions intervention (pre-intervention group). In the final 3 weeks all patients included received the intervention (intervention group). The main quantitative outcome measure was the level of SDM measured using the SDM-Q9 questionnaire at discharge (range 0–100). In addition, interviews with four patients of the intervention group were conducted and qualitatively analyzed.

**Results:**

Thirty-three patients were included in the pre-intervention group, 29 patients in the intervention group. The mean score of the SDM-Q9 in the pre-intervention group was 65.5 (SD 22.83) and in the intervention group 63.2 (SD 20.21), a not statistically significant difference. In the interviews, patients reported the three questions to be very useful. They used the questions mainly as a prompt and encouragement to ask more specific questions.

**Discussion:**

No difference in SDM was found between the two groups, possibly because of a small sample size. Yet the intervention appeared to be feasible and simple to use in an inpatient department. Further studies are needed to evaluate the impact of implementation of these three questions on a larger scale.

**Electronic supplementary material:**

The online version of this article (10.1186/s12884-018-1921-z) contains supplementary material, which is available to authorized users.

## Background

Shared decision making (SDM) is an important aspect of modern health care. Patients prefer to be involved in making their own medical decisions [1]. However, currently, patients reported not receiving enough information about options and corresponding advantages and disadvantages [2, 3]. In addition, patients’ preferences are often not taken into account when making health decisions [4]. In other words, patients are not provided with optimal personalized information [5], while these aspects are crucial in the process of SDM. Aside from an ethical imperative, SDM can result in better outcomes, such as increased understanding of their health condition, lower anxiety and greater compliance to treatment plans [6]. Many interventions have been designed and evaluated to improve SDM. These interventions can be provider-directed (e.g. training) [7] or patient-directed, such as patient decisions aids [8]. Decision aids are developed to improve patients’ knowledge about their medical condition and risk perception. Ultimately, it helps them in making an informed decision based on their personal preferences.

A simple patient-directed intervention to improve SDM was introduced by Shepherd and colleagues and showed promising results [9]. Patients visiting their general practitioner in an outpatient clinical setting were shown a 4-min video-clip, a pamphlet and a website and appeared to be successful in prompting participants to ask three questions in the consultation with their doctor. These questions were conducted from a consumer advocacy program ‘Patient first program’ in Western Australia and a health advise book ‘Smart health choices’ [10]. The goal of this program was to create three questions for patients stimulating healthcare providers to give patients the most optimal information and allow patients to make an informed medical decision. The three questions were:

1. What are my options/possibilities?
2. What are the benefits and harms of these options?
3. How likely are each of these benefits and harms to happen to me?

This study showed that 87% of the patients (*N* = 197) attending an outpatient family planning clinic and making a medical decision, asked all three questions. It provided patients with more suitable information about their options. It also improved patients’ involvement in decision-making. This study also showed that patients preferred making the primary decision and wanting to get all the information possible from their care provider.

Studies on SDM in obstetrics [11–13] indicated that women prefer to be more involved in medical decisions. Moreover, in the Netherlands, a 2016 interdisciplinary protocol for integral pregnancy care [14] introduced SDM as an important point of attention. In this protocol the three questions are mentioned as a tool to improve SDM. However, to the best of our knowledge none of the SDM interventions have been tested in an inpatient clinical setting. This is remarkable as it is common knowledge that patients admitted to an inpatient department are usually very dependent of the medical staff and control is an important factor for the patients comfort [15]. Patients frequently get mixed messages from different doctors while hospitalized [16] and most patients do not feel involved in decision-making while in hospital [17]. Given the simple nature of the ‘three questions’ intervention, we aimed to evaluate the feasibility and effectiveness on SDM of this intervention in an inpatient department. Additionally, as this is a pilot study, testing the feasibility of conducting a larger scale study on this topic is also an aim.

## Methods

### Design

This is a clinical pilot study evaluating the feasibility and effectiveness of the three questions intervention on SDM in a clinical inpatient setting. We carried out a mix-method study, in which we combined a quantitative pre- and postintervention study including qualitative in-depth interviews with a selection of participants from the intervention group.

### Setting and participants

All patients older than 18 years and admitted to the obstetrics inpatient unit of the Radboud university medical center in the Netherlands were asked to participate. These patients were hospitalized requiring tertiary care because of severe and complicated problems during their pregnancy or problems after childbirth. Non-Dutch speaking patients were excluded. Recruitment took place in June and July 2016. All patients admitted in the study period were informed and those willing to participate signed an informed consent form.

Total duration of the study was 6 weeks. In the first 3 weeks patients who participated did not receive the three questions intervention (pre-intervention group). In the last 3 weeks, all participating patients received the three questions intervention at admission (intervention group). The three questions were printed on a card, so patients could conveniently keep it with them.

Patients were encouraged to use these questions during ward rounds on the department. Typically, physicians (i.e. obstetricians and obstetrics trainees), did ward rounds on patients with pregnancy- related problems or complex postpartum. A hospital-based midwife did ward rounds on the majority of postpartum patients. An observer (SB) was present during ward rounds for the entire 6 weeks to measure the length of each consultation between physician or midwife and patient. To provide a complete and adequate description of the intervention we used the TIDieR checklist as a guideline, since this checklist has shown to be useful in adequate reporting of interventions [18].

Before commencing the study both an e-mail and presentation with information about the study and a presentation was provided to all medical staff (physicians, midwives, nurses). This included information about the study design, the ‘three question’ intervention, and the primary and secondary outcomes of the study. At admission all patients received an information pamphlet to inform them about the study. The three questions were not specifically mentioned in this pamphlet, as patients in the control group would be biased. One of the researchers (SB) then approached individual patients, and asked them to participate in this study. During the first 3 weeks patients in the pre-intervention group were asked to act as they normally would do and ask the questions they would normally ask during clinical ward rounds. During the last 3 weeks patients in the intervention group received the card with the three questions. One of the researchers (SB) gave information about the three questions and encouraged patients to use this card and its questions during daily ward rounds during their hospital stay. Medical staff was aware which patients received the intervention, because we had specific timeframes for the pre-intervention and intervention period (i.e. 3 weeks) and because of the visibility of the card. Physicians and midwives were instructed to perform their ward rounds as they would normally do.

### Data collection

Our primary outcome was the patient’s perceived level of SDM, as measured with the Shared Decision Making questionnaire (SDM-Q9). The SDM-Q9 contains nine items relevant to shared decision making, e.g. ‘My doctor made clear that a decision needs to be made.’ The items rate from 0 until 6 on a Likert scale (0 = completely disagree to 6 = completely agree) with a continuous scale and with a scoring range from 0 to 54. This questionnaire is widely used and translated in multiple languages [19–23]. A study of Rodenburg-Vandenbussche [21] showed a good acceptance, internal consistency and reliability of the Dutch version of the SDM-Q9. Patients of both the pre-intervention and intervention group were asked to complete the SDM-Q9 questionnaire at discharge. Patients, who were hospitalized for more than 1 week, filled out the questionnaire weekly. In addition, at inclusion patients were asked to complete a questionnaire with seven general background questions, such as age, educational level, ethnicity, reason for admission, number of pregnancies, duration of hospital stay and number of admissions to the ward during the current pregnancy. Also, they were asked about their preferences about their involvement in decision making and information provision, as was done in the study by Shepherd et al. [9].

Finally, we purposively selected four patients from the intervention group for an interview, to substantiate the interpretation of our results and to establish future recommendations for the three questions intervention. These interviews had an open and semi-structured character using an interview guide (see Additional file 1). Questions that were included were for example: ‘What did you think when you saw the questions for the first time?’ and ‘What do you think we want to accomplish with these questions?’. The interviews were performed during the last week of the intervention period

### Analysis

As we performed a pilot study to evaluate the feasibility of the use of the three questions in a clinical inpatient setting, we aimed at including 25 to 50 patients per group.

The participants’ demographics and background characteristics were analyzed using descriptive statistics. Using the Chi-squared test we calculated the statistically significant differences between the demographics of the patients and reason for admission were tested between the pre- intervention and intervention group. Differences in duration of hospital stay were calculated with an independent sample T-test, because of the numerical, continuous outcome. Because the SDM-Q9 questionnaire has an unfamiliar range (0-54), we rescaled these scores to a more practical range of 0-100 [20]. To study the difference between the two study groups on the SDM-Q9 questionnaire and the length of consultations during ward rounds we used an independent sample T-test for numerical, continuous outcomes. In case of statistically significant differences in baseline characteristics between the two study groups, we performed multivariate regression analyses to account for this difference. Statistically significance for all analyses was set at P<0.05. All analyses were done using Statistical Package for the Social Sciences (SPSS), version 22.

## Results

**Fig. 1:**
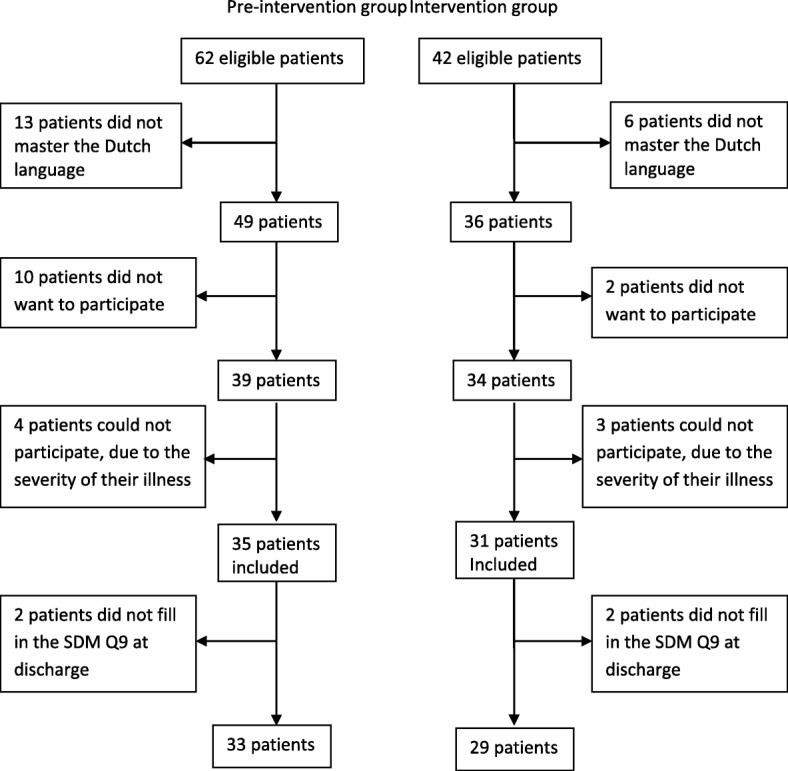
Recruitment flowchart

A total of 104 patients were eligible to participate in the study: 62 patients in the pre-intervention group and 42 patients in the intervention group (Fig. 1). Of the 62 eligible patients of the pre- intervention group, 13 patients did not speak the Dutch language adequately, 10 patients did not want to participate and four patients were not able to participate, due to the severity of their illness. Eventually, 35 patients were included. In the intervention group, six patients did not have an adequate level of the Dutch language, two patients did not want to participate and three patients were not able to participate, due to the severity of their illness. The intervention group finally contained of 31 participants. In both groups, two participants were excluded, because they had not completed the SDM-Q9 questionnaire at discharge. This resulted in 33 patients for the pre- intervention group and 29 patients for the intervention group.

Mean age of participating patients was 31 years old in the pre-intervention group and 32 years old in the intervention group (P=0.092) (Table 1). The majority of patients was Dutch (95.2%), had a high educational level (61.3%), and was admitted for the first time during the current pregnancy (79.0%) being pregnant of their first child (56.5%). Background characteristics were comparable between the two groups and did not differ statistically significant. The reason for admission was the only variable to be statistically significant different (*P*=0.005) between the two groups. The main reason for admission in the pre-intervention group was a pregnancy-related problem (81.8%), whereas the main reason in the intervention group was a postpartum medical problem (51.7%). These results are presented in Table 1.

**Table 1:** Characteristics of study sample (pre-intervention group *n* = 33, intervention group *n* = 29)

		Pre-intervention group N %		Intervention group N %		*P* value
Age						
18–24		1	3.0	2	6.9	
25–30		13	39.4	8	27.6	
30–35		13	39.4	6	20.7	
> 35		6	18.2	13	44.8	0.092
Ethnicity						
Dutch		32	97.0	27	93.1	
Other		1	3.0	2	6.9	0.365
Education level						
Medium - Low		14	42.5	10	34.4	
High		19	57.5	19	65.6	0.522
Reason for admission						
Problem in pregnancy		27	81.8	14	48.3	
Postpartum		6	18.2	15	51.7	0.005
Hospitalizations in this pregnancy						
First		26	78.8	23	79.3	
Second		4	12.1	4	13.8	
Third		0	0	2	6.9	
Fourth		3	9.1	0	0	0.176
Total pregnancies						
First		17	51.5	18	62.1	
Second		11	33.3	9	31.0	
Third		4	12.1	1	3.5	
Fourth		1	3.0	1	3.5	0.620

The duration of hospitalization was statistically significant different (*P*=<0.001, 95% CI 0.72-7.56) between the two groups. The pre-intervention group had a mean hospitalization period of 8.2 days (SD 9.33) compared to 4.1 days (SD 2.41) in the intervention group. Two patients in the pre-intervention group had a long hospital stay, namely 43 and 30 days. When excluding these patients from the analysis the difference between the two groups was still statistically significant (*P*= <0.001, 95% CI 0.031-4.601). Patients in both groups had similar involvement and information preferences (Table 2). The majority of patients in both groups preferred the doctor to make the decision, but only after the doctor explicitly considered the patient’s needs and priorities (pre-intervention 60.6% and intervention group 51.7%). For information preferences, 20 patients (60.6%) in the pre-intervention group and 11 patients (37.9%) in the intervention group preferred as many details as possible about the choice that needed to be make.

**Table 2:** Involvement and information preference concerning SDM compared between pre-intervention group and intervention group

	Pre-intervention group N %		Intervention group N %		P value
Involvement preferences					
The doctor should make the decisions using all that’s known about the treatments	2	6.1	2	6.9	
The doctor should make the decisions but strongly consider my needs and priorities	20	60.6	15	51.7	
The doctor and I should make the decisions together on an equal basis	4	12.1	5	17.2	
I should make the decisions, but strongly consider the doctor’s opinion	6	18.2	7	24.1	
I should make the decisions using all I know or learn about the treatments	1	3.0	0	0	0.800
Information preferences					
Prefer as many as details as possible	20	60.6	11	37.9	
I want only information needed to care for myself properly	5	15.2	8	27.6	
I want additional information only if it is good news	0	0	0	0	
I want as much information as possible, good and bad	8	24.2	10	34.5	0.194

The mean score of the SDM-Q9 in the pre-intervention group was 65.5 (SD 22.86) and in the intervention group 63.2 (SD 20.21). This was not a statistically significant difference (*P*=0.64). Also when correcting for the reason of admission and duration of hospitalization, using multivariate analysis, no statistically significant difference (*P*=0.41) in SDM-Q9 scores between the two groups was found.

With respect to the secondary outcome, multivariate analysis showed a significant difference in duration of consultation between the two groups: 6min 33 sec in the pre-intervention group versus 7min 26 sec in the intervention group (*P*=0.03) (Table 3).

**Table 3:** Statistics of SDM-Q9 score and duration of consultation during ward rounds, compared between pre- intervention group and intervention group

	N	Mean (SD)	Mean difference	95% confidence interval
Score of SDM-Q9 (range 0–100)				
Pre-intervention group	54	65.47 (±22.86)		
Intervention group	31	63.20 (±20.21)	4.780	−7.272 – 11.801
Duration of consultation (seconds)				
Pre- intervention group	135	392.94 (±278.60)		
Intervention group	71	445.73 (±254.35)	38.550	− 128.947 – 23.364

Six patients participating in the intervention group were approached for an interview and four of them agreed. All four patients believed that the questions were a method to gain more information from the physician. The three questions were primarily used as a prompt and reminder to ask more specific questions. Patients reported that the three questions were not always applicable. Before receiving the intervention, treatment plans were usually already known, most of the three questions were already answered and patients were often waiting to recover or to go into labor while hospitalized. However, if a new problem occurred while in the ward the questions were useful immediately. A selection of quotes can be seen in Table 4.

**Table 4:** Comments from in-depth interviews with four patients concerning SDM

I think the questions are very good and handy. They are general questions, so applicable in different situations, as a reminder to ask more questions.	
These questions are to accomplish better communication between doctor and patient, I think. So patients will get more information about the treatment options and so forth. I believe doctors should in any case give answers to these questions, but I understand that this is not always the case. Also you never know exactly what the patient wants to know, so these questions are very helpful for the patient to ask more specific questions.	
I didn’t use the three questions literally in the consultation with the doctor. I used the three questions to form more specific questions, which were more applicable to my situation.	
These three questions are not so much applicable while you’re admitted in the hospital and the whole plan is already known. While admitted the patient is usually waiting. There are very little changes [during the hospital stay]. Therefore you have few questions. If a new situation occurs, then I would definitely use the three questions to gain more information. I also think it is convenient that the patient has the questions in advance, so you can use them immediately, if necessary.	

## Discussion

This study introduced the ‘three questions’ as an intervention to improve SDM in a clinical inpatient setting. This study showed no difference in SDM between patients in an inpatient clinical setting with and without using the three questions intervention. The duration of the consultations during ward rounds was statistically significant higher in the intervention group (53 seconds). From in-depth interviews we learned that patients thought that the questions were very convenient to gain more information from care providers. The ‘three questions’ intervention prompted patients to ask more, but also more specific questions. This suggests that the three questions stimulated the awareness of patients to ask questions to their medical team. The card with the three questions seems a simple and feasible method to improve SDM. The implementation of this intervention to daily care routines seemed easy and did not encounter any problems. Effortless implementation on a larger scale is therefore likely, if shortcomings of this study are taken into account.

To the best of our knowledge, this is the first study to evaluate a SDM intervention in an inpatient obstetric department. Strength of the study is the addition of in-depth interviews to the quantitative data. The qualitative data did provided some more insight into the quantitative results, although only four interviews were conducted because of the small sample size of this pilot study, in which saturation was reached.

However, some limitations of the study should be mentioned as well. First, this study had a small sample size, which might have resulted in the lack of significant findings on our main outcome measure. In general this is a common phenomenon in pilot and feasibility studies. This was also the reason for choosing a non-randomized design. Nevertheless, this limitation should be taken into account when interpreting the results. Second, different healthcare providers consulted patients that participated in this study. Physicians were lead care providers for patients admitted for pregnancy- related problems, whereas hospital-based midwives led the ward rounds for most patients with postpartum problems. Although physicians and hospital-based midwives in the Netherlands both get training in communication with patients, the approach, techniques and focus during conversations with patients could be different. Finally, both groups were not comparable because there was a significant difference in reason for admission between groups (i.e. problem in pregnancy vs. postpartum problem).

Furthermore, blinding of patients and care providers was not possible, because of the visibility of the card containing the three questions during clinical ward rounds. It was attempted to minimize bias by keeping patients in the pre-intervention group oblivious for the purpose of the study. Finally, care providers were aware of the aim of the study. This could have affected the conversation between them and the patient, which can result in a response bias.

Shepherd et al [9] developed and evaluated the ‘three questions’ intervention in an outpatient setting. Also, they did not specifically evaluate the impact of this intervention on the process of SDM. The qualitative part of our study showed that the three questions were primarily used as a reminder to ask more questions, and to form new questions better suitable for the patient’s particular situation. The questions were not literally used during ward rounds. This in contrast to Shepherd’s study [9]. This study was designed to encourage patients to literally use the questions in the consultation with their care provider. Also opposed to Shepherd et al. [9]*,* we found a statistically significant increase of consultation duration when patients used the three questions. However, in our study patients had either a physicians or a midwife as the lead care provider during ward rounds, which might explain this difference. Also, the number of consultations per patient might affect the length of consultations. We did not collect that information in this study.

During the interviews, we found that the three questions were not always suitable for a clinical inpatient setting. For example, in this hospital patients are often first evaluated in an outpatient assessment unit before admitted to the ward. We noticed that some of the care providers already provided patients with a lot of information at admission about the reason for admission and treatment plan, in which also patients’ view on these things are discussed. It could be argued that the three questions intervention needs to be implemented immediately at admission in the outpatient assessment unit, where most of the information about the reason for admission and the management plan is provided and deliberated. This is similar to the results of other studies reporting that SDM was not or less possible because of the specific clinical setting [1, 24].

Because this study is conducted in an obstetrics inpatient unit, all participants included were women. Studies of LaCousiere [25] and Nussbaum [26] showed that women have a say in over 80% of the health-care decisions they make and are usually the primary decision maker. On the contrary, men are more likely to prefer a paternalistic approach of physicians compared to women. The same accounts for older and less educated patients [1]. In our study, we found that most patients wanted to make the primary decision about their medical situation. Therefore, our patient group seems to be a representative group of women.

We have chosen to exclude patients who do not speak Dutch adequately. However, this might be a group of patients that could particularly benefit from this intervention. Patients having a language barrier are more prone of being miss- or under informed while hospitalized, especially in patient-physician communication about diagnosis, risks and emergency situations [27]. A recent meta-analysis [13] showed that SDM decision aids have a greater positive effect on disadvantaged groups, compared to a normal population. Especially their knowledge, participation, decisional conflict and self-efficacy increases when using decision aids. With respect to the three questions intervention, this intervention could be translated into different languages and accompanied with visual sign to increase SDM in patients with a language barrier.

## Conclusion

This pilot study was a brief evaluation of the three questions intervention on SDM in an inpatient clinical setting. We recommend that more attention should be given to studies that focus on the improvement of SDM in an inpatient clinical setting, as this is lacking in current literature. The ‘three questions’ intervention seems simple and feasible. It might be at least helpful in prompting patients to be more actively involved in decision-making and ask more questions to medical staff. Further research is needed to study the impact of this intervention on SDM on a larger scale and in different settings.

## Additional file

Additional file 1: Interview guide, here translated in English was used during interview with four purposively selected patients from the intervention group were, to substantiate the interpretation of our results and to establish future recommendations for the three questions intervention. (DOCX 14 kb)

## Abbreviations

SD: Standard deviation
SDM: Shared decision making
SDM-Q9: Shared decision making questionnaire 9 SPSS = statistical package for the social sciences
TIDieR: Template for intervention description and replication
