# Supplementary material for: Improving shared decision-making in a clinical obstetric ward by using the three questions intervention, a pilot study
Source: BMC Pregnancy Childbirth. 2018 Jul 4;18:283. doi: 10.1186/s12884-018-1921-z (PMC6031181; doi:10.1186/s12884-018-1921-z)
Supplement: Supplementary file 1 — Interview guide, here translated in English was used during interview with four purposively selected patients from the intervention group were, to substantiate the interpretation of our results and to establish future recommendations for the three questions intervention. (DOCX 14 kb) [file 12884_2018_1921_MOESM1_ESM.docx]

**INTERVIEW GUIDE**

- What comes first to mind when you see these [three] questions?
- What do you think is the goal of using these three questions?
- What do you think of the questions?
  - Prompt: Are they always applicable?
  - Prompt: Should they be used every ward round?
  - Prompt: If so, why and when? If not, why not?
- How would you change the questions in order to make them applicable?
- How do you feel about ‘having to’ ask these questions during ward round?
  - Prompt: Is it hard? Is it easy?
  - Prompt: Is it easy to find a moment to ask the questions?
- Do you know what shared decision making is?
  - Prompt: If so, what do you think of it?
  - Prompt: When should it be applied and when not?
  - Prompt: And what do you think of shared decision making when admitted to the hospital?
- What do you think of the shared decision making questionnaire that you have completed?
